# Reliability, biological variability, and accuracy of multi-frequency bioelectrical impedance analysis for measuring body composition components

**DOI:** 10.3389/fnut.2024.1491931

**Published:** 2024-12-03

**Authors:** David P. Looney, Erica A. Schafer, Christopher L. Chapman, Riana R. Pryor, Adam W. Potter, Brandon M. Roberts, Karl E. Friedl

**Affiliations:** ^1^United States Army Research Institute of Environmental Medicine (USARIEM), Natick, MA, United States; ^2^Maximize Human Performance, LLC, Framingham, MA, United States; ^3^CoachMePlus, Buffalo, NY, United States; ^4^Oak Ridge Institute for Science and Education (ORISE), Oak Ridge, TN, United States; ^5^Center for Research and Education in Special Environments, Department of Exercise and Nutrition Sciences, University at Buffalo, Buffalo, NY, United States

**Keywords:** body composition, BMI, FMI, DXA, body water, BIA

## Abstract

**Introduction:**

Bioelectrical impedance analysis (BIA) systems are gaining popularity for use in research and fitness assessments as the technology improves and becomes more affordable and easier to use. Multifrequency BIA (MF-BIA) may improve accuracy and precision using octopolar contacts for segmental analyses.

**Purpose:**

Evaluate reliability, biological variability, and accuracy of component measures (total body water, mass, and composition) of commercially available MF-BIA system (InBody 770, Cerritos, California, USA).

**Methods:**

Fourteen healthy military-age adults were assessed by MF-BIA in duplicate on five laboratory visits across 3 weeks (10 measures each). Participants were evaluated at the same time of day after refraining from strenuous exercise (> 48 h), alcohol consumption (> 24 h), and caffeine, nicotine, and food (> 10 h). Systematic error (test–retest reliability) and biological variability (day-to-day reliability) were summarized by intraclass correlation coefficient (ICC) values determined for body mass (fat, fat-free, total) and body water (extracellular, intracellular, total). Body composition measurements derived from BIA on the second visit were also tested for accuracy compared to dual-energy x-ray absorptiometry (DXA).

**Results:**

Test–retest reliability was very high for all measurements of whole-body water and mass (ICC ≥ 0.999) and high for regional body water and mass (ICC 0.973–1.000). Biological variability was observable with very minor differences between tests (same day) for total and regional body water (0.0–0.2 L) and total and regional body mass measurements (0.0–0.2 kg); while between day differences were slightly higher (0.0–0.5 L and 0.1–0.7 kg). Compared to DXA, the MF-BIA whole-body measures showed an offset in %BF (Bias −4.0 ± 2.8%; Standard error of the estimate (SEE), 2.6%), an overprediction for total body fat-free mass (Bias 2.8 ± 2.1 kg; SEE 2.2 kg) and an underprediction of total body fat mass (Bias −2.9 ± 2.0 kg; SEE 1.9 kg).

**Conclusion:**

Under controlled conditions with fit and healthy men and women, this MF-BIA system has high methodological reliability and demonstrates stable day-to-day measurements of major body composition components. Previously reported ~3% body fat offset compared to criterion methods was again confirmed. Precision of the InBody 770 shows consistency and supports further testing of this specific device as a new military standards method and suitability across a wider range of %BF.

## Introduction

Bioelectrical impedance (BIA) is an appealing technology for practical and affordable everyday assessment of body composition across many domains, including preventive medicine, athletics, and assessing compliance with military body fat standards. Interest in electrical impedance technologies has soared in the past two decades as evidenced by the number of publications in this field (1). The cost and time associated with measuring body composition with BIA is considerably less than the criterion methods such as dual-energy x-ray absorptiometry (DXA) or the previously used hydrodensitometry (2). In 1994, the National Institutes of Health convened a Technology Assessment meeting on BIA that was reviewed by an expert extramural panel. The panel concluded that “BIA provides a reliable estimate of total body water (TBW) under most conditions [and] it can be a useful technique for body composition analysis in healthy individuals…” (3). However, the leading researchers involved in this early consensus also noted that BIA is affected by factors such as body position, hydration and prandial state, skin temperature, and recent physical activity (4, 5). Since this time, considerable improvements in the BIA technology have been made, which increases the interest in using BIA to replace current reliance on anthropometric estimates of adiposity (e.g., body mass index (BMI), skinfold thicknesses, body circumferences) with a method that is equitable across sexes and has better accuracy and precision at the individual level. Compared to anthropometric methods such as body circumferences and skinfold thicknesses, a method such as BIA which does not involve assessment of different fat sites in men and women can also be viewed as more equitable, and the ability of a user to obtain their own reliable measurements without contact with an observer increases user acceptability. Biophysically-based strategies such as BIA also hold out the promise of greater precision free from observer error; the US Army briefly used the valid and accurate Durnin & Womersley equations for skinfold thicknesses but then abandoned the method because of the substantial interobserver variation when applied at scale (i.e., the entire US Army) (6).

The original concept of BIA was to use stature^2^/resistance of a cylindrical model of the human body to predict body water and fat-free mass. These assessments were based on measurement of resistance to 50 kHz of current applied across the length of the connected series of cylinders (i.e., between hand and foot on one side of the body). This frequency passed through cell membranes and assessed total body water (7). Subsequent advances include octopolar assessments with two sensors on each hand and foot, and analysis across multiple frequencies (MF-BIA) or frequency sweep (bioelectrical impedance spectroscopy, BIS) (1, 8). Most devices are standardized for standing or prone body positions and studies have generally incorporated a standardized period of adjustment with postural changes (e.g., >15 min). Further improvements such as segmental analyses may reduce error from limb length assumptions, and most commercially available systems standardize posture (usually standing) and limb placement. The estimations of regional fluid and cell mass is still largely empirical and Ward has highlighted the need for a mechanistic understanding of impedance and phase angle measurement associations (9).

As part of a large-scale body composition survey of the U.S. Marine Corps, Potter et al. (10) determined that a commercially available BIA system provided similar results to DXA when a + 3% body fat correction was applied across a range of adiposity in free living conditions. However, this +3% body fat correction has yet to be cross-validated in another population of healthy, physically active, military-age women and men. Recently, the U.S. Army, Army National Guard, and Marine Corps have acquired and distributed BIA devices to be used by service members and reservists (11, 12). Given the importance of body composition standards in these occupations to physical readiness, the reliability and accuracy of these modern technologies must be evaluated for mass use beyond research applications. Furthermore, while BMI has historically been relied upon for identifying overweight and obese individuals, its usefulness compared to other methods such as the use of percent body fat has been called into question (13). Therefore, understanding the reliability and accuracy of modern BIA devices serves to help a broad range of the population and, with further testing, could replace BMI in clinical weight management.

In the present study, we assessed “best performance” using reliability and accuracy metrics of the technology under optimal controlled laboratory conditions to estimate percent body fat (%BF) and body cell mass (intracellular fluid and solids). Subsequent assessments can then consider the most important factors that must be controlled outside of the laboratory to ensure meaningful measurements. In this study, we tested a MF-BIA device that has been widely used in field research for %BF estimations. Furthermore, we examined accuracy of the MF-BIA compared to full body DXA, including segmental estimations of body composition, and we considered device and biological variability with repeated measurements on five separate days for each participant.

Current technology and prediction models may have improved enough to be able to justify the use of BIA for practical and affordable everyday assessments in preventive medicine, obesity prevention, fitness guidance, and for assessment of compliance with military body fat standards. This could replace current reliance on anthropometric estimates of adiposity (e.g., BMI, % body fat predictive equations, circumference tape methods) with a method that is equitable across sexes and has better accuracy and precision at the individual level. Accurate estimates of body measurements are also valuable for calculating derived metrics such as the various body mass indices. While BMI has historically been relied upon for identifying overweight and obese individuals, its usefulness compared to other methods such as the use of percent body fat has been called into question (13).

## Methods

### Design

We performed a repeated measures design study to evaluate the test–retest reliability and day-to-day variability of measurements of regional and whole-body water, mass, and composition from a commercially available system (InBody 770, Cerritos, California, USA). Study participants completed BIA tests in duplicate on five laboratory visits scheduled over 19 ± 6 days. Test–retest reliability was examined by comparing measurements between duplicate tests on each visit while day-to-day variability was assessed using data collected across the five visits. Additionally, BIA-derived body mass and composition measurements collected on the second visit were compared against criterion data measured by DXA. We also evaluated whether adding the +3% correction to BIA-derived %BF from Potter et al. (10) improved accuracy and concordance with DXA measurements.

### Participants

Fourteen healthy military-age adults (age, 24 ± 6 years old; height, 172 ± 8 cm; body mass, 72.4 ± 15.9 kg) participated in the current study including 3 women (age, 21 ± 3 years old; height, 167 ± 7 cm; body mass, 62.4 ± 9.9 kg) and 11 men (age, 25 ± 6 years old; height, 173 ± 8 cm; body mass, 75.2 ± 16.4 kg). The fourteen participants included 11 active-duty U.S. Army Soldiers and 3 civilian men. Participants were recruited from the Natick Human Research Volunteer (HRV) Pool, U.S. Army Natick Soldier System Center (NSSC) active-duty military personnel, active-duty population located at other military organizations, as well as the federally and non-federally employed civilian population. Participants were required to be between 18 and 44 years old, at least recreationally active (aerobic or resistance exercise for at least 30 min on at least 2 days per week), and free of any musculoskeletal injuries, illnesses, or medical conditions that compromise the ability to exercise. Participants gave their voluntary written informed consent after a briefing on the purpose of the study and potential risks. This study was approved by the Institutional Review Board (IRB) at the U.S. Army Medical Research and Development Command (MRDC; Fort Detrick, MD, USA). Investigators adhered to Department of Defense Instruction 3216.02 and 32 CFR 219 on the use of volunteers in research.

### Procedures

Participants arrived at the laboratory in the same physical training uniform (e.g., shorts, t-shirt, socks, compression garment) at the same time of day (0700) after refraining from strenuous exercise (> 48 h) and alcohol consumption (> 24 h) as well as avoid caffeine, nicotine, food, over the counter medications, and dietary supplements (> 10 h). All metallic items (jewelry, etc.) were removed prior to testing. There was no attempt to control for menstrual cycle or contraceptive hormone use in the women. To ensure proper hydration, participants were asked to drink at least 500 mL water the night before as well as the morning of each visit. Participants provided a urine sample that was assessed for hydration status via urine specific gravity (USG) (<1.030) using a standard refractometer. No additional water was consumed within 1 h of the start of body composition testing. Height was measured using a laboratory stadiometer on the first visit.

Bioelectrical impedance analysis (BIA) was conducted using a commercially available system (InBody 770, Cerritos, California, USA). The InBody 770 system uses a multi-frequency assessment method (1, 5, 50, 250, 500 and 1,000 kHz) with proprietary algorithms apparently based on a sum of segments approach (14). For each test, the participant stood barefoot on the footplate with heels on the rear sole electrodes for an initial body mass measurement. Participants stood still for the duration of the BIA assessment with arms positioned straight and away from the body, thighs not in contact with one another, and hands gripping the hand electrode so that the thumb was placed on the oval electrode and the four fingers wrapped the surface of the bottom hand electrode. In addition to whole body measurements, we recorded the regional measurements reported by the InBody 770 system, which include arms (left and right arms combined), legs (left and right legs combined), and trunk. We also reported the remainder or difference between each whole-body measurement and the sum of its associated arm, leg, and trunk measurements.

On the second visit only, study participants were assessed via DXA (iDXA, GE Lunar, Madison, Wisconsin, USA). Participants laid still in the supine position within the outlined assessment area of the DXA table for a whole-body scan that lasted ~10 min. Similar to BIA, we recorded whole body and regional measurements (arms, legs, trunk, and remainder) for the DXA; these regional assessments were based on the default regions of interest in the software. All DXA scans were performed by the same cross-trained team members and required DXA training and certification to meet our rigorous laboratory credentialing standard. The coefficient of variation for same day repeated measures of percent body fat with the GE iDXA has been shown to be 1.06% (15).

### Statistical analyses

All data were analyzed using R (Version 4.3.3; R Foundation for Statistical Computing; Vienna, Austria) (16) and reported as mean ± standard deviation (SD) unless specified otherwise. For each outcome, we determined the intraclass correlation coefficient (ICC) (17) for test–retest reliability by partitioning out variance components using a linear mixed-effects model (18) that included random effects of visit within participant on intercepts. We compared body composition measurements from both BIA tests on the second visit against the criterion DXA using a linear mixed-effects model that included random effects of participant on intercepts. The agreement between BIA and DXA was evaluated quantitatively based on the bias (mean difference, BIA − DXA), standard deviation (SD) of differences, standard error of the estimate (SEE), Pearson correlation coefficient (r), and concordance correlation coefficient (CCC). We also included Bland–Altman plots with 95% limits of agreement (95% LoA) to visually depict the agreement between the two systems with the *x*-axes represented as the mean of paired measurements (19, 20).

## Results

Regional and whole-body water components measured by BIA had exceptional test–retest reliability (ICC ≥ 0.973) (Table 1). Test–retest reliability of body mass measurements was nearly perfect for all compartments and regions (ICC ≥ 0.995) except for fat mass of the remainder, which was still excellent (ICC = 0.949) (Table 2). Test–retest reliability was also nearly perfect for BIA-measured percent body fat as well as BMI, FMI, and fat free mass index (FFMI) (ICC ≥ 0.998) (Table 3).

**Table 1:** Test–retest reliability and variability of regional and whole-body water measurements (L) by bioelectrical impedance analysis (BIA).

				Variability between				
Compartment	Region	Mean	SD	Participant	Day	Test	Range	ICC
Intracellular	Arms	3.2	0.8	0.8	0.0	0.0	0.2 ± 0.1	0.999
	Legs	8.7	1.6	1.6	0.1	0.0	0.2 ± 0.1	1.000
	Trunk	12.8	2.5	2.5	0.1	0.1	0.4 ± 0.1	0.999
	Remainder	2.9	0.7	0.7	0.1	0.1	0.4 ± 0.2	0.985
	Whole	27.5	5.3	5.3	0.2	0.1	0.7 ± 0.3	1.000
Extracellular	Arms	1.9	0.5	0.5	0.0	0.0	0.1 ± 0.0	0.999
	Legs	5.0	0.9	0.9	0.1	0.0	0.2 ± 0.1	1.000
	Trunk	7.4	1.3	1.3	0.1	0.1	0.3 ± 0.1	0.998
	Remainder	1.7	0.4	0.4	0.1	0.1	0.3 ± 0.1	0.973
	Whole	16.0	2.9	2.9	0.2	0.1	0.6 ± 0.2	0.999
Total	Arms	5.1	1.3	1.3	0.1	0.0	0.3 ± 0.1	0.999
	Legs	13.7	2.5	2.5	0.1	0.0	0.4 ± 0.1	1.000
	Trunk	20.2	3.8	3.8	0.2	0.1	0.6 ± 0.2	0.999
	Remainder	4.6	1.0	1.0	0.2	0.1	0.6 ± 0.3	0.991
	Whole	43.6	8.1	8.1	0.5	0.2	1.3 ± 0.5	1.000

ICC, intraclass correlation coefficient for test–retest reliability within day; Range, difference between maximum and minimum measurement within each individual across the ten tests; Remainder, Whole – (Arms + Legs + Trunk); SD, standard deviation.

**Table 2:** Test–retest reliability and variability of regional and whole-body mass measurements (kg) by bioelectrical impedance analysis (BIA).

				Variability between				
Compartment	Region	Mean	SD	Participant	Day	Test	Range	ICC
Fat	Arms	1.4	1.1	1.1	0.1	0.1	0.3 ± 0.1	0.997
	Legs	3.8	1.8	1.8	0.2	0.1	0.5 ± 0.3	0.998
	Trunk	6.9	4.5	4.5	0.3	0.1	0.9 ± 0.3	0.999
	Remainder	1.2	0.3	0.2	0.2	0.1	0.4 ± 0.5	0.949
	Whole	13.3	7.4	7.3	0.5	0.2	1.5 ± 0.6	0.999
Fat-Free	Arms	6.6	1.7	1.7	0.1	0.0	0.3 ± 0.1	0.999
	Legs	17.6	3.2	3.2	0.2	0.0	0.5 ± 0.2	1.000
	Trunk	26.1	4.9	4.9	0.3	0.1	0.8 ± 0.3	0.999
	Remainder	9.3	1.9	1.9	0.2	0.1	0.7 ± 0.4	0.996
	Whole	59.6	11.1	11.1	0.6	0.2	1.8 ± 0.7	1.000
Total	Arms	8.0	2.3	2.2	0.1	0.1	0.3 ± 0.1	1.000
	Legs	21.5	4.1	4.1	0.3	0.1	0.8 ± 0.4	1.000
	Trunk	32.9	8.1	8.1	0.3	0.1	0.9 ± 0.3	1.000
	Remainder	10.4	2.0	2.0	0.3	0.1	0.9 ± 0.4	0.995
	Whole	72.9	15.9	15.9	0.7	0.0	1.7 ± 0.7	1.000

ICC, intraclass correlation coefficient for test–retest reliability within day; Range, difference between maximum and minimum measurement within each individual across the ten tests; Remainder, Whole – (Arms + Legs + Trunk); SD, standard deviation.

**Table 3:** Test–retest reliability and variability of key bioelectrical impedance analysis (BIA) measurements.

			Variability between				
Measurement	Mean	SD	Participant	Day	Test	Range	ICC
Body fat (% body mass)	17.6	7.4	7.3	0.6	0.3	1.9 ± 0.9	0.998
Body mass index (kg/m^2^)	24.5	4.0	4.0	0.2	0.0	0.6 ± 0.2	1.000
Dry lean mass (kg)	16.0	3.0	3.0	0.2	0.1	0.5 ± 0.2	0.999
Extracellular water (% TBW)	36.8	0.6	0.5	0.2	0.0	0.5 ± 0.2	0.994
Fat mass index (kg/m^2^)	4.5	2.3	2.3	0.2	0.1	0.5 ± 0.2	0.999
Fat-free mass index (kg/m^2^)	20.0	2.6	2.6	0.2	0.1	0.6 ± 0.3	0.999
Skeletal muscle mass (kg)	33.9	6.9	6.9	0.3	0.1	1.0 ± 0.4	1.000
Visceral Fat Area (cm^2^)	52.7	34.2	34.1	2.5	1.1	7.0 ± 2.9	0.999

ICC, intraclass correlation coefficient for test–retest reliability within day; Range, difference between maximum and minimum measurement within each individual across the ten tests; Remainder, Whole – (Arms + Legs + Trunk); SD, standard deviation; TBW, total body water.

**Figure 1:**
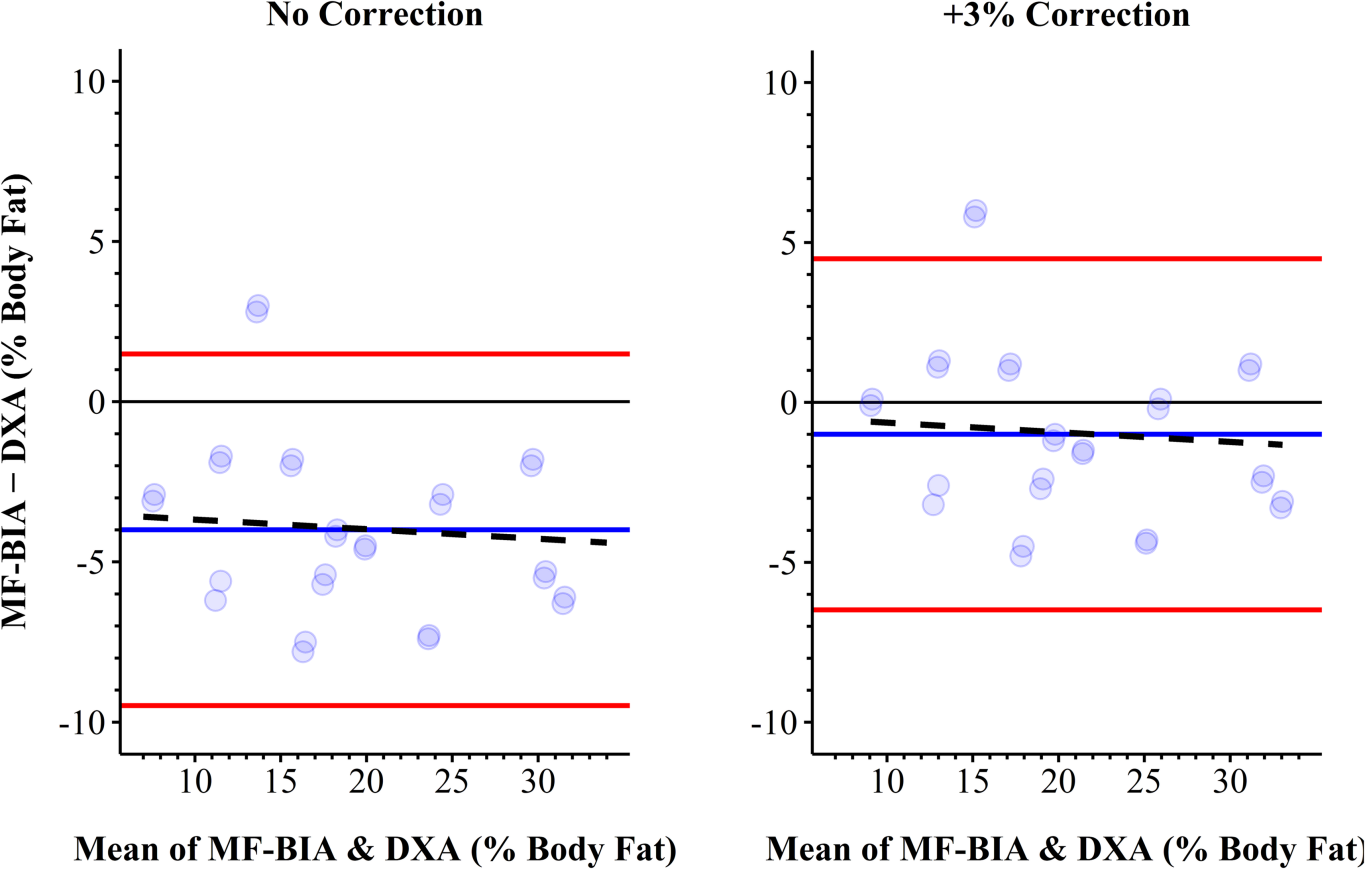
Bland–Altman plots of agreement between percent body fat measured by MF-BIA and DXA with no correction and + 3% correction (10) to MF-BIA. Black dashed line, linear regression line of best fit; Blue solid line, bias; DXA, dual-energy x-ray absorptiometry; MF-BIA, Multi-frequency Bioelectrical Impedance Analysis; red solid lines, 95% limits of agreement.

**Figure 2:**
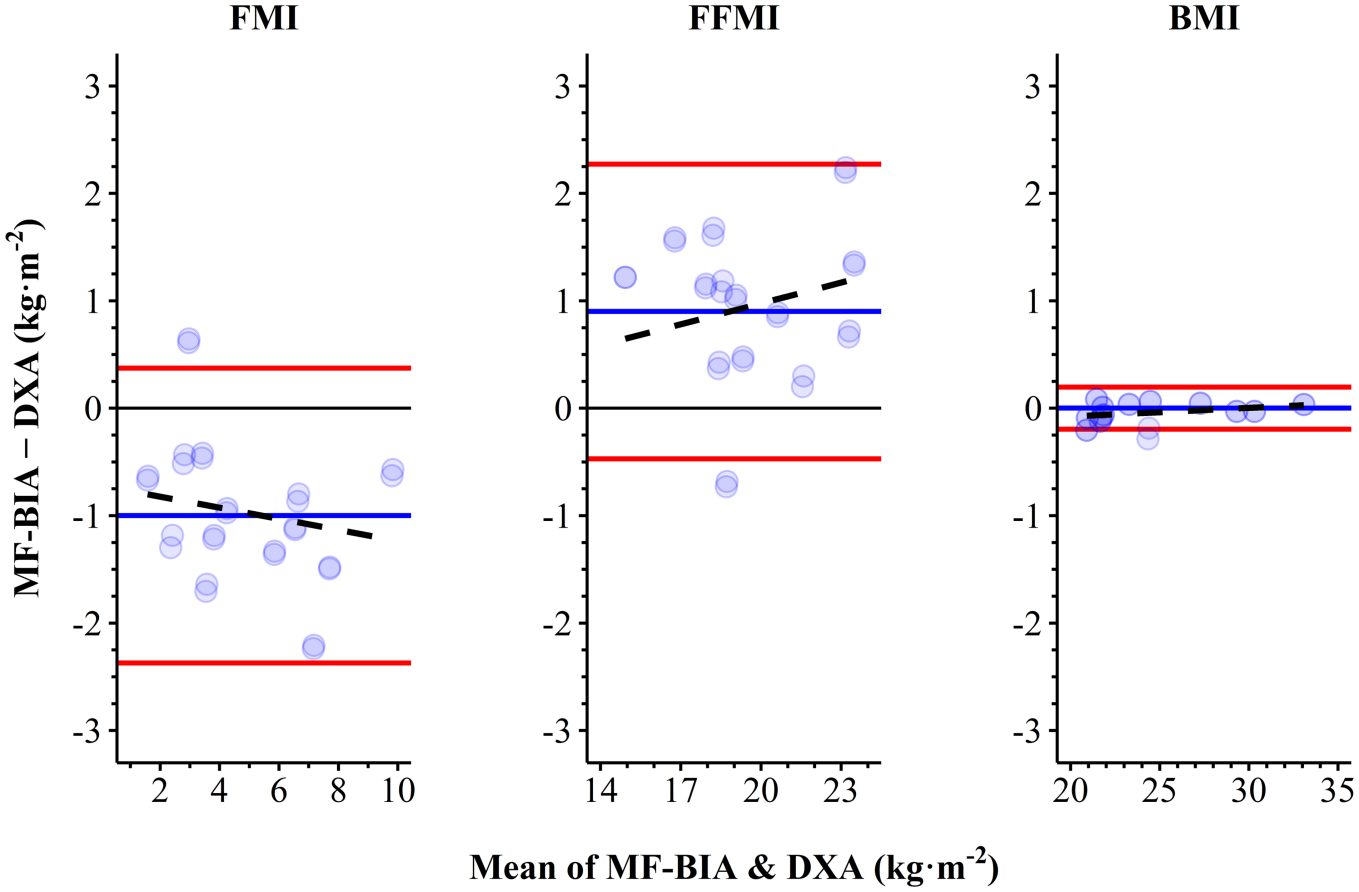
Bland–Altman plots of agreement between fat mass index (FMI), fat-free mass index (FFMI), and body mass index (BMI) measured by BIA and DXA. Black dashed line, linear regression line of best fit; Blue solid line, bias; DXA, dual-energy x-ray absorptiometry; MF-BIA, Multi-frequency Bioelectrical Impedance Analysis; red solid lines, 95% limits of agreement.

When compared to DXA, BIA typically measured lower regional fat, fat-free, and total mass except for fat-free mass measured at the trunk (Bias, 0.4 kg) and all mass measured for the remainder (Bias ≥0.3 kg) (Table 4). Percent body fat was consistently lower when measured by BIA versus DXA but strongly correlated (Bias ± SD, −4.0 ± 2.8%; SEE, 2.6%; r, 0.932; CCC, 0.819) (Figure 1). Adding the 3% body fat correction to BIA measurements from Potter et al. (10) increased both accuracy and concordance (Bias ± SD, −1.0 ± 2.8%; SEE, 2.6%; r, 0.932; CCC, 0.920). Agreement between BIA and DXA was good for FMI (Bias ± SD, −1.0 ± 0.7 kg∙m^2^; SEE, 0.6 kg∙m^2^; *r*, 0.961; *p* < 0.001; CCC, 0.886) and FFMI (Bias ± SD, 0.9 ± 0.7 kg∙m^2^; SEE, 0.7 kg∙m^2^; *r*, 0.957; *p* < 0.001; CCC, 0.896) but nearly perfect for body mass index (Bias ± SD, 0.0 ± 0.1 kg∙m^2^; SEE, 0.1 kg∙m^2^; *r,* 1.000; *p* < 0.001; CCC, 1.000) (Figure 2).

**Table 4:** Accuracy of bioelectrical impedance analysis (BIA) mass measurements (kg) relative to dual-energy x-ray absorptiometry (DXA).

Compartment	Region	Bias ± SD (kg)	SEE (kg)	Bias ± SD (%)	*r*	*p*	CCC
Fat	Arms	−0.4 ± 0.6	0.7	−44.9 ± 46.8	0.805	< 0.001	0.731
	Legs	−1.5 ± 1.3	1.0	−33.0 ± 26.5	0.838	< 0.001	0.644
	Trunk	−1.3 ± 1.1	1.0	−25.3 ± 36.1	0.973	< 0.001	0.935
	Remainder	0.3 ± 0.3	0.3	24.2 ± 21.6	0.169	0.534	0.063
	Whole	−2.9 ± 2.0	1.9	−22.3 ± 18.4	0.966	< 0.001	0.896
Fat-Free	Arms	−0.4 ± 0.5	0.4	−6.0 ± 7.1	0.968	< 0.001	0.930
	Legs	−2.5 ± 1.8	1.2	−12.6 ± 7.0	0.922	< 0.001	0.730
	Trunk	0.4 ± 2.3	2.4	1.4 ± 8.8	0.876	< 0.001	0.863
	Remainder	5.3 ± 1.5	1.4	80.2 ± 11.7	0.676	0.006	0.033
	Whole	2.8 ± 2.1	2.2	4.9 ± 3.7	0.980	< 0.001	0.947
Total	Arms	−0.9 ± 0.9	0.8	−10.6 ± 8.3	0.923	< 0.001	0.859
	Legs	−4.0 ± 2.0	1.3	−16.6 ± 5.8	0.949	< 0.001	0.681
	Trunk	−0.8 ± 2.3	2.3	−3.0 ± 6.8	0.957	< 0.001	0.950
	Remainder	5.6 ± 1.6	1.4	72.7 ± 11.2	0.686	0.005	0.039
	Whole	−0.1 ± 0.3	0.3	−0.1 ± 0.4	1.000	< 0.001	1.000

CCC, concordance correlation coefficient; *p*, *p*-value for Pearson’s correlation coefficient; *r*, Pearson’s correlation coefficient; SD, standard deviation; SEE, standard error of the estimate.

## Discussion

Our study demonstrates the high test-rest reliability and relatively low day-to-day variability of the InBody 770 BIA system when measuring common regional and whole-body water, mass, and composition measurements in healthy military-age adults across five controlled laboratory visits. When compared to DXA, BIA tended to measure lower values for arm, leg, and trunk mass as well as lower fat mass and higher fat-free mass for the whole body. Although BIA-derived percent body fat was systematically lower than when measured by DXA, adding the +3% body fat correction from Potter et al. (10) improved accuracy and concordance. Esco et al. (21) found that a closely related system from the same manufacturer (InBody 720) also underestimated percent body fat by 3.33% and overestimated fat free mass (FFM) by 2.12 kg in college female athletes when compared with DXA (21). Mecherques-Carini et al. (22) replicated our findings in a recent study by also finding an approximate 3% bias in body fat percentage as measured by BIA with a different commercially-available, segmental multifrequency BIA device with eight electrodes (MC-780-MA, Tanita Corporation, Tokyo, Japan) compared to DXA in young adults following an overnight fast. Another study comparing a SECA device (SECA 515) to iDXA also demonstrated an underestimation of fat mass and overestimation of fat-free mass (23).

Previous studies have also shown the InBody 770 tends to underestimate %BF (3.0 to 3.4%) while overestimating fat-free mass (2.2–2.7 kg) (10, 24–27). This appears to be a systematic error related to the predictive software. The lower arm, leg, and trunk masses measured by BIA versus DXA could be attributed to the different methodology used by each system for segmentation of body regions. Full-body DXA scans are generally separated into regions of interest automatically based on positioning of anatomical landmarks on a two-dimensional reconstructed image of the individual (28) whereas BIA estimates regional measurements based on empirical equations that incorporate sex, height, and body mass (29). Consequently, the proprietary algorithms employed by the systems examined in this study may also use different anatomical landmarks for separating body regions. Additionally, it is possible that the empirical equations used by the InBody 770 rely on inaccurate assumptions of body proportions for a modern population of healthy, physically active, military-age adults. This could be determined by a follow-up study comparing disagreements in BIA and DXA-derived body mass measurements between groups of individuals with extreme body types (high vs. low armspan-to-height ratio, etc.). Ultimately, what is made abundantly clear by the consistently higher remainder measured by the InBody 770 for each mass compartment is that this BIA system attributes considerably less mass to the arms, legs, and trunk than the DXA system. BMI and waist circumference were important predictors of the variance between MF-BIA and DXA with an earlier version of the InBody octopolar system (8).

There are several methodological considerations of the present study that warrant discussion. First, this study was designed to maximize internal validity and experimental control. Depending on the measurement, we demonstrated excellent reliability with BIA measurement in this tightly controlled experiment. These results may not be applicable to other types of BIA systems. Further research is required to assess how reliability is impacted in free-living applications such as for military standards and in fitness facilities, or by circadian rhythm influences over the time of day. While we recruited both women and men for the present study, we are limited by the small number of women (*n* = 3) who participated and who are also underrepresented in the military services.

To our knowledge, this is the first study to evaluate test–retest reliability of the InBody 770 BIA system over five visits within a relatively short period of time (19 ± 6 days). The consistency of the body water, mass, and composition measurements within individuals supports the use of the InBody 770 for longitudinal monitoring applications. An uncontrolled field study with Inbody measurements on separate days will provide a snapshot of biological variability produced by prandial, exercise, and hydration factors which were tightly controlled in this study. In this study, there was no attempt to control for phase of the menstrual cycle with potential changes in total body water; however, the biological variability in this limited sample of women was not markedly different than the ICC for the men.

## Conclusion

In tightly controlled conditions InBody 770 BIA system provides exceptionally reliable measurements of common regional and whole-body water, mass, and composition measurements in healthy miliary-age adults under controlled laboratory conditions. Users can add the +3% body fat correction from Potter et al. to their InBody measurements to obtain closer results to DXA.

## Data Availability

The datasets presented in this article are available by the authors upon reasonable request.
